# Relationship between angiogenic growth factors and atherosclerosis in renal transplantation recipients: a cross-sectional study

**DOI:** 10.1590/1516-3180.2024.0120.05062024

**Published:** 2024-10-21

**Authors:** Melahat Çoban, Beyza Algul Durak, Mine Sebnem Karakan

**Affiliations:** I Assistant Professor, Department of Nephrology, Ankara Bilkent City Hospital, Ankara, Turkey.; II Department of Nephrology, Ankara Bilkent City Hospital, Ankara, Turkey.; III Professor, Department of Nephrology, Yıldırım Beyazıt University, Ankara, Turkey.

**Keywords:** Atherosclerosis, Angiopoietin-2, Vascular endothelial growth factors, Renal resistive index., Renal transplant recipients., Renal failure

## Abstract

**BACKGROUND:**

Accelerated development of atherosclerosis has been observed in renal transplant recipients (RTRs). Angiopoietin-2 (Ang-2) and vascular endothelial growth factor (VEGF) are vascular enzymes that play important roles in vascular development and angiogenesis.

**OBJECTIVE:**

This study aimed to investigate the relationship between Ang-2 and VEGF and atherosclerosis in RTRs.

**DESIGN AND SETTING:**

This study was conducted at Ankara City Hospital, Turkey.

**METHODS:**

This cross-sectional study included 36 (37.5%) female and 60 (62.5%) male RTRs. All findings were compared with those of 70 healthy controls. Ultrasonographic measurements of the carotid artery intima-media thickness (CA-IMT) and renal resistive index (RRI) were used as indicators of atherosclerosis.

**RESULTS:**

Log_10_ Ang-2, log_10_ VEGF, CA-IMT, and RRI levels were significantly higher in patients than in healthy controls. No significant differences were detected in CA-IMT and RRI between those with log_10_ Ang-2 ≥ 3.53 pg/mL and those with < 3.53 pg/mL. No significant differences were detected in CA-IMT and RRI between those with log_10_ VEGF ≥ 1.98 pg/mL and those with < 1.98 pg/mL. No correlation was detected between log_10_ Ang-2 and log_10_ VEGF, CA-IMT, or RRI.

**CONCLUSIONS:**

Increased serum angiogenic growth factor levels and increased atherosclerosis development were detected in RTRs compared to healthy individuals. No relationship was observed between angiogenic growth factors and atherosclerosis. This may be due to the decreased synthesis and effect of angiogenic growth factor receptors synthesized from atherosclerotic plaques due to atherosclerosis, which improves after renal transplantation.

## INTRODUCTION

Increased cardiovascular disease (CVD) has been observed in patients with chronic kidney disease (CKD), including renal transplant recipients (RTRs). Renal transplantation is the preferred treatment modality for patients with end-stage renal failure, providing significant survival and quality of life advantages over long-term dialysis. Although RTRs are highly susceptible to infection and have an increased tendency to develop malignancies, CVD is the main cause of mortality among RTRs. A 5-fold increase in cardiovascular (CV) mortality was detected one year after renal transplantation compared with that in the age-matched control group. In RTRs, the risk factors for CVD development are divided into two categories: traditional and non-traditional. Traditional risk factors are divided into two categories: immutable (age, sex, and inheritance) and variable (smoking, hyperlipidemia, hypertension, obesity, diabetes mellitus, physical activity, and stress). Non-traditional risk factors include transplantation and treatment (immunosuppressive agents, graft rejection, and viral infection) and chronic rejection (anemia, volume load, hyperhomocysteinemia, oxidative stress, secondary hyperparathyroidism, and microinflammation).^1^

Atherosclerosis is characterized by chronic, unrecoverable inflammation and cholesterol accumulation in the vascular walls of medium and large arteries. Neovascularization, unstable plaque formation, and rupture play important roles in its development. The presence of atherosclerosis can be assessed using renal Doppler ultrasonography with carotid artery intima-media thickness (CA-IMT) as a reproducible, noninvasive, and simple method. Increased CA-IMT may be regarded as an indicator of increased risk of CV events. The development of atherosclerosis, as determined by CA-IMT, is more common in RTRs than in the normal population.^2^

Determining the renal resistive index (RRI) using Doppler ultrasonography allows for the assessment of renal resistance and renal arteriolar damage. Values of ≥ 0.80 are indicative of adverse renal function and increased mortality,^3^ but a decreased RRI may be a sign of renal stenosis. Chudek et al. reported that RRI is a sensitive but not specific marker for graft dysfunction.^4^ Radermacher et al. reported that a high RRI may be used as a strong indicator of graft loss.^3^ Kramann et al. reported that RRI obtained in the first 6 months after the transplantation failed to predict graft failure; however, RRI obtained 12-18 months may be useful in predicting long-term graft outcomes.^5^ Shimizu et al. reported that RRI may be used as a reliable marker for atherosclerosis.^6^ Calabia et al. found a significant relationship between RRI and CA-IMT and argued that this relationship could provide useful data on micro- and macrovascular damage.^7^ For this reason, measuring RRI with ultrasonography can be considered an easy and non-invasive method to detect graft functions and the presence of atherosclerosis.

The balance between pro- and anti-angiogenic factors regulates angiogenesis, a process that requires an interaction between endothelial cells, extracellular matrix, and surrounding cells, mediated by a set of growth factors, their receptors, and intracellular signals. The angiopoietin (Ang)-Tie ligand receptor system consists of 2 receptor tyrosine kinases (Tie-1 and Tie-2) and four ligands (Ang-1, Ang-2, Ang-3, and Ang-4). Ang-2 is a ligand of Tie-2 receptor, a second-class vascular-specific receptor tyrosine kinase. Ang-2 is stored in granules called Weibel-Palade bodies (WPB) in endothelial cells. The Ang/Tie system tightly controls the endothelial phenotype during angiogenesis. Loss of vascular integrity, vascular leakage, and neutrophil migration occur due to Ang-2 effects. Therefore, it is considered a pro-inflammatory factor. Ang-2 is expressed at active vascular remodeling and angiogenesis sites, and is induced by various cytokines, including vascular endothelial growth factor (VEGF). Ang-2 acts as an agonist that stimulates angiogenesis by causing vascular destabilization in the presence of VEGF. Ang-2 competitively antagonizes Tie-2 phosphorylation in the absence of VEGF, causing vascular regression and endothelial cell death.^8^ Thus, Ang-2 and VEGF act synergistically to form a stable and functional microvasculature. High serum Ang-2 levels are detected in patients with diabetes mellitus, arteriosclerosis, acute coronary syndrome, arterial hypertension, and acute renal injury.

As important regulators of angiogenesis, lymphangiogenesis, lipid metabolism, and inflammation, the VEGF family comprises of heparin-binding proteins that play a role in atherosclerosis and other CVDs development. The VEGF family consists of five gene products in humans, three of which regulate blood vessel growth (VEGF-A, VEGF-B, placental growth factor), and two of which regulate lymphangiogenesis (VEGF-C and VEGF-D). Additionally, there are three VEGF receptors: VEGF receptor 1 (VEGFR1) (FLT1 gene); VEGFR2 (KDR gene), which is mainly expressed in vascular endothelial cells; and VEGFR3 (FLT4 gene), which is expressed in lymphatic endothelial cells. The primary VEGF expression site is in the CV system (endothelial cells, angioblasts, and pericytes). However, it is also expressed in several other cell types during inflammation and hypoxia. VEGF receptors are distributed in vascular smooth muscle cells, osteoblasts, cardiomyocytes, myofibroblasts, neurons, and various tumor cells.^9^ VEGF also plays roles in endothelial cell function, physiological angiogenesis (formation of blood vessels during tissue revascularization), and pathological angiogenesis (as a marker of ischemic diseases, inflammation, and microvascular occlusion). Increased VEGF levels induce endothelial cell proliferation and vascular permeability. VEGF also plays a dual role in atherosclerosis. However, it sometimes acts as a mitogen via re-endothelialization, causing harmful effects by preventing the repair of endothelial lesions that induce atherogenesis.

Although many studies have been conducted with stage 1-5 patients with CKD, few studies have investigated the development of atherosclerosis in RTRs and the relationship between growth factors and atherosclerosis.

## OBJECTIVE

This study aimed to investigate the relationship between Ang-2 and VEGF and atherosclerosis, as determined by CA-IMT and RRI in RTRs.

## METHODS

### Patient selection

This cross-sectional study was conducted with 96 RTRs, including 36 female (37.5%) and 60 male (62.5%), who were followed up in the Organ Transplantation Polyclinic. The patient group was compared with a control group, which comprised 70 healthy volunteers with a similar distribution of age and sex. The exclusion criteria were as follows: refusal to participate in the study, active infection and malignancy, peripheral vascular disease, previous history of cardiac intervention (coronary angiography, valvular replacement, cardiac pacemaker), or history of heart disease detected echocardiographically (atrial fibrillation, left ventricular systolic dysfunction [LVEF] < 50%). The study was explained to the participants and approved by the Ethics Committee of Ankara City Hospital on March 20, 2024 (TABED 2-24 68)

### Laboratory measurements

Venous blood samples taken from all participants after an 8-12 h overnight fasting were centrifuged at 4 °C for 10 min, and the supernatants were stored at -80 °C. Serum creatinine, total cholesterol (TC), high-density lipoprotein cholesterol (HDL-C), triglyceride, calcium (Ca), phosphate (P), 25 hydroxy(OH)vitamin(Vit) D_3_ (25(OH)VitD_3_), and parathyroid hormone (PTH) levels were analyzed according to standard methods. Low-density lipoprotein cholesterol (LDL-C) levels were calculated using the Friedewald’s formula. Ang-2 (Elabscience, Shanghai, China) and VEGF (Novex Life Technologies, Thermo Fisher Scientific, Germany) levels were measured using commercial enzyme-linked immunosorbent assay kits. For all parameters, %CV was < 10%; analytical range and analytical sensitivity values were 45.78-2970 pg/mL and 26.12 pg/mL for Ang-2 and 30.14-1989 pg/mL and 17.64 pg/mL for VEGF, respectively.

### Measurement of CA-IMT

The right and left common carotid arteries (CCA) were visualized using a high-resolution B-mode ultrasonography (USI) device (Siemens, USA), using a 5-10 MHz linear probe. Measurements were performed in the supine position while the patient’s neck was angled approximately 20° to the contralateral side at three points: right and left CCA, bifurcation, and the first 2 cm segment of the internal carotid artery. CA-IMT was measured by evaluating the posterior wall. CA-IMT was determined by longitudinal examination of the distance between the vascular lumen echogenicity and media/adventitia echogenicity. Each measurement was repeated three times, and the average of the left and right measurements was calculated.

### Measurement of RRI

Measurements were performed using a high-resolution B-mode ultrasonography (USI) device (Siemens, Saint Paul, Minnesota, United States) and a 5 MHz vector probe. The examination was performed in the supine and/or prone position in transverse and longitudinal sections of the kidney from the straight segment of the renal artery near the hilus on the arcuate arteries (at the corticomedullary junction) or interlobar arteries (adjacent to medullary pyramids). No measurements were obtained from the accessory arteries. The RRI was calculated using the following formula: (maximum systolic flow rate - end-diastolic flow rate)/ maximum systolic flow rate. The RRI was determined at least three times for both kidneys and averaged to obtain the mean RRI value for each patient. The RRI values were calculated as the average of all determinations in the two kidneys. The normal range is 0.50-0.70. A high RRI (> 0.8) in native kidneys is associated with adverse CV events. The intraobserver coefficients of variance of RRI were 4.4% for the main renal artery and 5.1% for the interlobar artery.

### Statistical analyses

The normality assumptions of the variables were examined using the Kolmogorov-Smirnov test. The Mann-Whitney U test was used to compare continuous variables that did not show a normal distribution between the groups, and the independent samples t-test was used to compare variables that had a normal distribution. The relationships between categorical variables were examined using chi-square or Fisher’s exact tests, and the relationships between continuous variables were examined using Spearman’s RHO correlation analysis. Multivariate regression analysis was used to determine the parameters predicting log_10_ VEGF and log_10_ Ang-2 variables. The IBM SPSS version 25 (IBM Corp., Armonk, NY, USA) was used for all analyses, with a significance level of P < 0.05.

## RESULTS

### Patients and healthy individuals

The study included 36 (37.5%) female and 60 (62.5%) male RTRs who had a mean age of 43.51 ± 11.51 years. The patients were compared with a group of 70 age- and gender-matched healthy controls who had a mean age of 44.94 ± 10.69 years. The mean time after transplantation was 40.8 ± 2.31 months. Nine (9.4%) patients had diabetes mellitus and 81 (84.4%) had hypertension. Mean creatinine, PTH, Ca, P, and 25(OH)VitD_3_ were 1.24 ± 0.46 mg/dL, 102.21 ± 97.24 pg/mL, 9.28 ± 0.50 mg/dL, 3.20 ± 0.64 mg/dL, and 16.85 ± 7.22 ng/mL, respectively. TC, LDL-C, HDL-C, and triglyceride values were 186.01 ± 38.72 mg/dL, 105.00 ± 33.93 mg/dL, 49.01 ± 13.06 mg/dL, and 157.44 ± 87.84 mg/dL, respectively. Mean log_10_ Ang-2 was 3.48 ± 0.18 pg/mL and log_10_ VEGF was 1.88 ± 0.48 pg/mL. The mean CA-IMT was 0.91 ± 0.34 mm and RRI was 0.66 ± 0.06. Creatinine (P < 0.001), PTH (P < 0.001), triglyceride (P < 0.001), log_10_ Ang-2 (P = 0.009), log_10_ VEGF (P = 0.029), CA-IMT (P < 0.001), and RRI (P < 0.001) were significantly higher in the patients than in the healthy controls, and estimated glomerular filtration rate (eGFR) (P < 0.001), 25(OH)VitD_3_ (P < 0.001), TC (P = 0.004), and HDL-C (P < 0.001) were significantly lower (**Table 1**).

**Table 1:** Clinical data, demographic characteristics, and laboratory values of patients and healthy controls

	Patients (n = 96) Mean ± SD / n (%)	Healthy control group (n = 70) Mean ± SD / n (%)	P
Age (years)	43.51 ± 11.51	44.94 ± 10.69	0.029
Female/Male	36 (37.5%)/60 (62.5%)	39 (55.7%) /31 (44.3%)	0.026
BMI (kg/m^2^)	27.47 ± 5.82	25.53 ± 5.51	0.003*
Time since transplant (months)	40.8 ± 2.31		
Diabetes mellitus Hypertension	9 (9.4%) 81 (84.4%)		
Cyclosporine-MMF/MNa-steroid Tacrolimus-MMF/MNa-steroid Everolimus-tacrolimus-steroid	10 (10.4%) 77 (80.2%) 9 (9.3%)		
Creatinine (mg/dL)	1.24 ± 0.46	0.82 ± 0.11	< 0.001*
eGFR (mL/min/1.73 m^2^)	63.69 ± 20.84	88.10 ± 13.28	< 0.001**
PTH (pg/mL)	102.21 ± 97.24	43.38 ± 15.33	< 0.001*
Ca (mg/dL)	9.28 ± 0.50	9.25 ± 0.33	0.810*
P (mg/dL)	3.20 ± 0.64	3.19 ± 0.50	0.801*
25(OH)VitD_3_ (ng/mL)	16.85 ± 7.22	27.54 ± 14.10	< 0.001*
TC (mg/dL)	186.01 ± 38.72	205.47 ± 41.00	0.004*
LDL-C (mg/dL)	105.00 ± 33.93	124.27 ± 39.15	0.007*
HDL-C (mg/dL)	49.01 ± 13.06	60.26 ± 10.70	< 0.001*
Triglyceride (mg/dL)	157.44 ± 87.84	105.51 ± 35.46	< 0.001*
Log_10_ Ang-2 (pg/mL)	3.48 ± 0.18	3.47 ± 0.10	**0.009**
Log_10_ VEGF (pg/mL)	1.88 ± 0.48	1.79 ± 0.34	**0.029**
CA-IMT (mm)	0.91 ± 0.34	0.67 ± 0.07	< 0.001*
RRI	0.66 ± 0.06	0.63 ± 0.04	< 0.001*

*Mann Whitney U test;

** Independent samples t-test.

BMI = body mass index; MMF = mycophenolate mofetil; Mna = mycophenolate sodium; eGFR = estimated glomerular filtration rate; PTH = parathyroid hormone; Ca = calcium; P = phosphate; 25(OH)VitD_3_ = 25 hydroxy(OH)vitamin(Vit) D_3_; TC = total cholesterol; LDL-C = low density lipoprotein cholesterol; HDL-C = high density lipoprotein cholesterol; VEGF = vascular endothelial growth factor; Ang-2 = angiopoietin-2; CA-IMT = carotid artery intima-media thickness; RRI = renal resistivity index.

### Relationship between average growth factor values and atherosclerosis

The mean log_10_ Ang-2 was 3.53 pg/mL, and no significant differences were detected between those with log_10_ Ang-2 ≥ 3.53 pg/mL and those with log_10_ Ang-2 < 3.53 pg/mL regarding PTH, Ca, P, 25(OH)VitD_3_, CA-IMT, and RRI (**Table 2**).

**Table 2:** Comparison of patient characteristics according to median of log_10_ angiopoietin-2

	Log10 Ang-2 < 3.53 (n = 51) Mean ± SD	Log10 Ang-2 ≥ 3.53 (n = 45) Mean ± SD	P
Time since transplant (months)***	128.24 ± 73.39	110.61 ± 61.38	0.200
eGFR (mL/min/1.73 m^2^)****	62.92 ± 21.18	64.56 ± 20.65	0.704
PTH (pg/mL)****	102.78 ± 113.89	101.56 ± 75.34	0.280
Ca (mg/dL)****	9.15 ± 0.42	9.42 ± 0.55	0.006
P (mg/dL)****	3.30 ± 0.63	3.09 ± 0.64	0.117
25(OH)VitD_3_ (ng/mL)***	16.39 ± 8.10	17.43 ± 5.97	0.499
CA-IMT (mm)****	0.93 ± 0.35	0.88 ± 0.33	0.621
RRI****	0.65 ± 0.06	0.66 ± 0.06	0.365

***Mann Whitney U test;

****t-test in independent samples

SD = standard deviation; Ang-2 = Angiopoietin-2; eGFR = estimated glomerular filtration rate; PTH = parathyroid hormone; Ca = calcium; P = phosphate; 25(OH)VitD_3_ = 25 hydroxy(OH)vitamin(Vit) D_3_; CA-IMT = carotid artery intima-media thickness; RRI = renal resistivity index.

The mean log_10_ VEGF was 1.98 pg/mL, and no significant differences were detected between those with log_10_ VEGF ≥ 1.98 pg/mL and those with Log_10_ VEGF < 1.98 pg/mL regarding PTH, Ca, P, 25(OH)VitD_3_, CA-IMT, and RRI (**Table 3**).

**Table 3:** Comparison of patient characteristics according to median of log_10_ vascular endothelial growth factor

	Log10 VEGF < 1.98 (n = 48) Mean ± SD	Log10 VEGF ≥ 1.98 (n = 48) Mean ± SD	P
Time since transplant (months)***	122.90 ± 80.67	117.19 ± 53.56	0.791
eGFR (mL/min/1.73 m^2^)****	67.42 ± 19.06	59.96 ± 22.05	0.079
PTH (pg/mL)****	82.90 ± 87.64	121.52 ± 103.29	0.102
Ca (mg/dL)****	9.18 ± 0.39	9.37 ± 0.58	0.128
P (mg/dL)****	3.25 ± 0.51	3.16 ± 0.76	0.654
25(OH)VitD_3_ (ng/mL)***	17.00 ± 7.34	16.69 ± 7.18	0.838
CA-IMT (mm)****	0.94 ± 0.35	0.88 ± 0.32	0.272
RRI****	0.65 ± 0.06	0.67 ± 0.07	0.122

***Mann Whitney U test;

****t-test in independent samples

VEGF = vascular endothelial growth factor; SD = standard deviation; eGFR = estimated glomerular filtration rate; PTH = parathyroid hormone; Ca = calcium; P = phosphate; 25(OH)VitD_3_ = 25 hydroxy(OH)vitamin(Vit) D_3_; CA-IMT = carotid artery intima-media thickness; RRI = renal resistivity index.

### Relationship between growth factors and atherosclerosis

**Figure 1:**
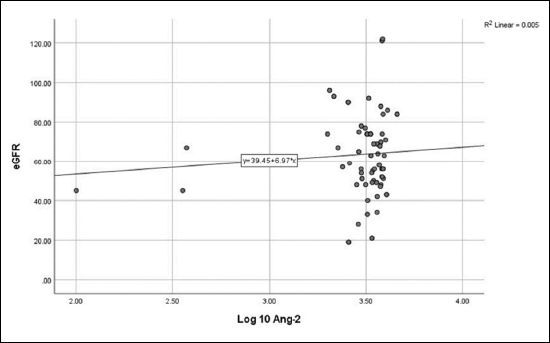
Relationship between angiopoietin-2 and estimated glomerular filtration rate (r = 0.073, P = 0.479).

**Figure 2:**
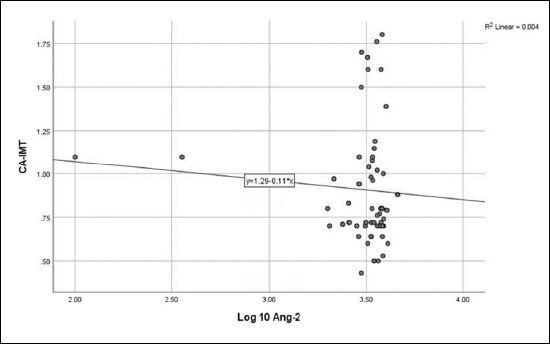
Relationship between angiopoietin-2 and carotid artery intima-media thickness (r = -0.067, P = 0.533).

**Figure 3:**
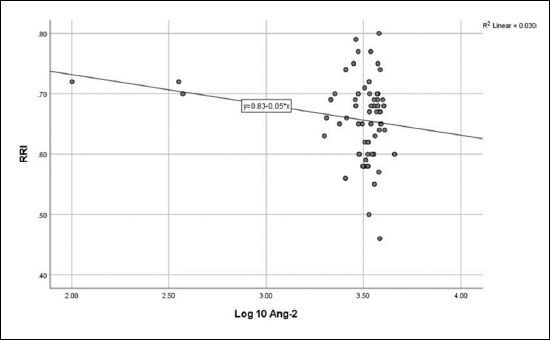
Relationship between angiopoietin-2 and renal resistivity index (r = - 0.172, P = 0.097).

**Figure 4:**
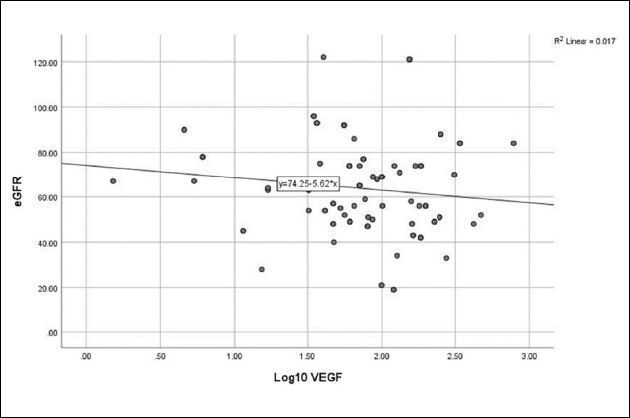
Relationship between vascular endothelial growth factor and estimated glomerular filtration rate (r = -0.130, P = 0.205).

**Figure 5:**
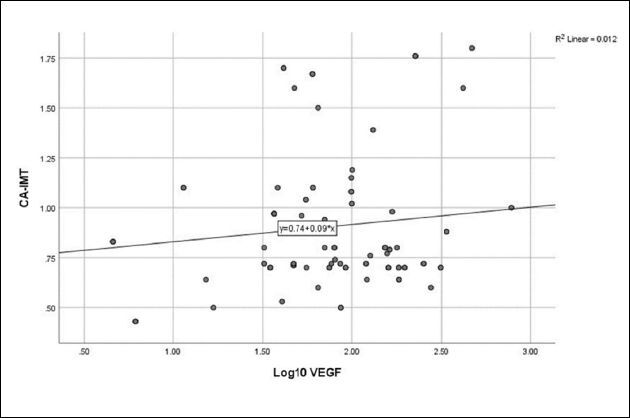
Relationship between vascular endothelial growth factor and carotid artery intima-media thickness (r = 0.111, P = 0.299).

**Figure 6:**
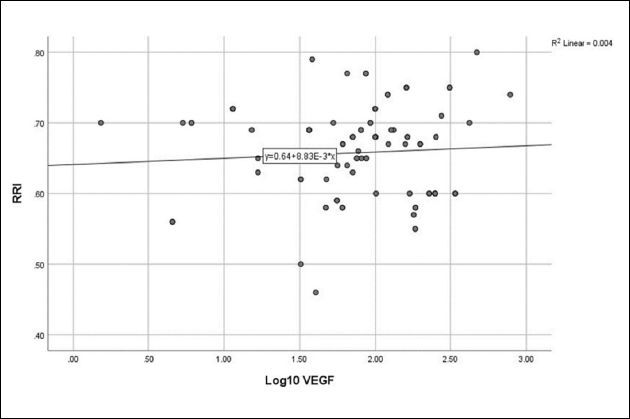
Relationship between vascular endothelial growth factor and renal resistivity index (r = 0.067, P = 0.522).

No correlation was detected between log_10_ Ang-2 and eGFR (**Figure 1**), CA-IMT (**Figure 2**), or RRI (**Figure 3**). No relationship was detected between log_10_ VEGF and eGFR (**Figure 4**), CA-IMT (**Figure 5**), or RRI (**Figure 6**) (**Table 4**). No relationships were found in the multivariate analysis between log_10_ Ang-2 and log_10_ VEGF, eGFR, CA-IMT, or RRI (**Table 5**).

**Table 4:** Correlation between serum growth factors and atherosclerosis

	Log10 VEGF r	Log10 VEGF P	Log10 Ang-2 r	Log10 Ang-2 P
Time since transplant (months)	0.053	0.610	-0.117	0.260
eGFR (mL/min/1.73 m^2^)	-0.130	0.205	0.073	0.479
PTH (pg/mL)	0.134	0.194	0.014	0.890
Ca (mg/dL)	0.170	0.197	0.216	0.350
P (mg/dL)	-0.134	0.193	-0.070	0.498
25(OH)VitD_3_ (ng/mL)	0.016	0.879	0.102	0.338
CA-IMT (mm)	0.111	0.299	-0.067	0.533
RRI	0.067	0.522	-0.172	0.097

Mann Whitney U test; Spearman correlation test VEGF = vascular endothelial growth factor; Ang-2 = Angiopoietin-2; eGFR = estimated glomerular filtration rate; PTH = parathyroid hormone; Ca = calcium; P = phosphate; 25(OH)VitD_3_ = 25 hydroxy(OH)vitamin(Vit) D_3_; CA-IMT = carotid artery intima-media thickness; RRI = renal resistivity index.

**Table 5:** Association between serum angiogenic growth factors and atherosclerosis in multivariate analysis

	β	SE	P
**Log10 VEG**F			
eGFR (mL/min/1.73 m^2^)	0.000	0.002	0.868
CA-IMT (mm)	0.024	0.133	0.859
RRI	0.896	0.705	0.207
**Log10 Ang-2**			
eGFR (mL/min/1.73 m^2^)	0.000	0.001	0.707
CA-IMT (mm)	-0.053	0.068	0.437
RRI	-0.489	0.361	0.179

SE = standard error; VEGF = vascular endothelial growth factor; Ang-2 = Angiopoietin-2; eGFR = estimated glomerular filtration rate; CA-IMT = carotid artery intima-media thickness; RRI = renal resistivity index.

## DISCUSSION

Increased serum Ang-2 and VEGF levels were detected in RTRs compared to those in healthy individuals in our study. In addition, increased atherosclerosis development, as determined by CA-IMT and RRI, was found in RTRs compared to those in healthy individuals. No relationship was found between Ang-2 and VEGF and atherosclerosis.

Patients with CKD show increased atherosclerosis, as determined by CA-IMT; however, results regarding the effects of renal transplantation on atherosclerosis development are conflicting. In a study conducted with 178 RTRs, Yilmaz et al. reported an improvement in atherosclerosis 6 months after the transplantation and that the improvement was associated with the accompanying increase in eGFR, but the values were still higher when compared to healthy individuals.^10^ Nafar et al. reported that the CA-IMT value increased gradually 2, 4, and 6 months after the transplantation.^11^ Pinho et al. reported accelerated atherosclerosis development after renal transplantation.^12^ Junarta et al. detected no relationships between eGFR and atherosclerosis and observed increased atherosclerosis development determined by CA-IMT in RTRs.^13^ Basinatria et al. reported that the CA-IMT value was higher in young RTRs than in healthy individuals and that the development of subclinical atherosclerosis was observed concerning sex and cumulative calcitriol dose.^14^ Kasiske et al. reported an acceleration in the atherosclerotic process after the transplantation in RTRs who did not have atherosclerotic disease before the transplantation.^15^ Lindholm et al. reported that atherosclerotic complications were significantly higher in RTRs than in the normal population.^16^ Turkmen et al. found increased atherosclerosis, determined by CA-IMT, in RTRs when compared to healthy individuals, and this was associated with increased oxidative stress, expression of pro-inflammatory and prothrombotic molecules, and decreased endothelial repair ability.^17^ Increased atherosclerosis determined by CA-IMT was observed in RTRs compared to healthy individuals in the present study.

Renal dysfunction (anatomical and functional changes in the microcirculation of the kidney) implies increased RRI values because of a decreased number and area of postglomerular capillaries. An increased RRI is associated with the degree of renal impairment as a measure of increased microvascular tonus. Scarring in the kidneys causes a decrease in the area of intrarenal vessels, which causes an increase in intrarenal vascular resistance.^3^ Calabia et al. reported that the mean RRI value was 0.69 ± 0.08 and RRI values were significantly higher in patients with CKD.^7^ Shimizu et al. reported significant relationships between RRI and carotid atherosclerosis.^6^ Radermacher et al. reported that increased RRI value was associated with increased mortality in RTRs.^3^ Heine et al. reported that RRI was a complex integration of arterial compliance, pulsatility, and peripheral resistance in RTRs and was associated with subclinical atherosclerotic vascular damage and traditional CV risk factors; therefore, it is a marker of not only renal but also general vascular atherosclerosis.^18^ Akgul et al. reported a relationship between RRI and CV risk factors and atherosclerosis determined by CA-IMT in RTRs.^19^ Brennan et al. reported a significant relationships between RRI and traditional CV risk factors and subclinical atherosclerosis in renal transplantation.^20^ Köger et al. found a significant correlation between mean renal transplantation RRI and mean internal carotid artery RRI in RTRs and noted that RRI was associated with overall atherosclerosis. They also reported that traditional CV risk factors and markers of subclinical atherosclerosis were associated with elevated RRI in RTRs.^21^ Increased atherosclerosis determined by RRI was observed in RTRs compared with healthy individuals in the present study.

In the present study, increased serum Ang-2 levels were detected in RTRs compared with those in healthy individuals. David et al. reported an inverse relationship between serum Ang-2 levels and eGFR in patients with CKD, and showed that circulating Ang-2 levels increased in patients with stage 1-5 CKD and patients on dialysis, and also reported that Ang-2 levels increased shortly after nephrectomy in a group of 15 healthy renal donors and correlated with a decrease in eGFR. They noted that the high Ang-2 levels returned to normal 3 months after renal transplantation. Endothelial WBP, synthesized from activated endothelial cells in patients with CKD, is the primary source of Ang-2. WBP secretion is activated by physical damage (hypoxia and trauma), endogenous chemicals (reactive oxygen species, histamine, and serotonin), and proteins (thrombin, VEGF, etc.). As the only known inhibitor of WBP exocytosis, nitric oxide is decreased in patients with CKD.^22^ In vivo studies have shown that pharmacological inhibition of nitric oxide production increases endothelial WPB exocytosis. Increased Ang-2 levels may result from excessive WPB exocytosis because of decreased nitric oxide in patients with CKD.^23^ Yang et al. reported higher serum Ang-2 levels in peritoneal dialysis patients than in healthy individuals, suggesting that increased Ang-2 levels resulted from excessive WPB exocytosis because of decreased nitric oxide production. Ang-2 expression has also been detected in high glucose and tumor necrosis factor-alpha levels in dialysis patients in vitro.^24^

Tsai et al. showed an independent association between increased serum Ang-2 levels and all-cause mortality and adverse CV events in patients with CKD.^25^ David et al. reported a relationship between increased Ang-2 and increased CV mortality in patients with CKD.^26^ Iribarren et al. reported that increased serum Ang-2 levels may be associated with CV disease progression and could be used as a marker for CV events that might develop.^27^ Le et al. reported that Ang-2 was elevated in vascularized and rupture-prone human atherosclerotic plaques.^28^ El-Asrar et al. reported that Ang-2 was a significant independent risk factor for atherosclerosis because of its role in vascular dysfunction in patients with type 1 diabetes mellitus.^29^ Yang et al. reported a relationship between Ang-2 and atherosclerosis in peritoneal dialysis patients and that high Ang-2 levels would independently predict fatal and non-fatal CV events.^24^ Shroff et al. studied children undergoing dialysis treatment and found an association between increased serum Ang-2 levels and atherosclerosis as assessed by CA-IMT.^30^ Mayer et al. reported that Ang-2 showed an increase with advancing disease stage in patients with CKD, and caused increased atherosclerosis.^31^ David et al. reported that Ang-2 was a marker of atherosclerosis in patients with CKD and that Ang-2-induced endothelial activation had important roles in the pathogenesis of atherosclerosis.^22^ No relationships were found between Ang-2 and atherosclerosis determined by CA-IMT and RRI in RTRs in the present study. Ahmed et al. reported that Ang-2 inhibits atherosclerosis by limiting LDL oxidation over a nitric oxide-dependent pathway by stimulating the release of nitric oxide from endothelial cells.^32^ David et al. reported that elevated Ang-2 was an indicator of atherosclerosis in dialysis patients and that Ang-2 was not a marker of atherosclerosis in renal transplantation, attributing this to the disappearance of atherosclerotic changes after renal transplantation.^33^

In the present study, increased serum VEGF levels were observed in RTRs compared to healthy individuals. Blann et al. reported elevated serum VEGF levels in diabetic patients.^34^ Liu et al. suggested an increased serum VEGF levels in patients with CKD compared to healthy individuals probably due to decreased excretion because of decreased renal function or VEGF being closely associated with CKD pathogenesis.^35^ Nguyen et al. reported that VEGF was inversely correlated with eGFR in patients with diabetic stage 3-5 CKD and increased serum VEGF levels were detected when compared to healthy individuals.^36^ Pilmore et al. reported an increased serum VEGF levels in RTRs because of hypoxia and decreased renal blood flow in chronic rejection.^37^ Rintala et al. reported that VEGF ligands and receptors increased after renal transplantation in mice.^38^

The role of VEGF in the molecular mechanisms underlying atherosclerotic lesions remains controversial. Angiogenesis mediates plaque growth, promoting the influx of erythrocytes and inflammatory cells, resulting in plaque rupture and deterioration of atherosclerosis. Hypoxia and inflammation in atherosclerotic plaques trigger VEGF synthesis in macrophages. Felmeden et al. reported a relationship between high serum VEGF levels and endothelial damage/dysfunction and CV risk in hypertensive patients.^39^ Celletti et al. suggested that VEGF potentially enhanced the development of early atherosclerotic plaques and contributed to plaque destabilization and atherosclerosis deterioration.^40^ Inoue et al. noted that VEGF promoted the development of atherosclerosis by stimulating monocyte chemotaxis and plaque neovascularization.^41^ Celletti et al. showed that VEGF promotes atherosclerotic plaque formation in mice^40^ and Ohtani et al. showed that it stimulated the development of atherosclerosis through the infiltration of macrophages and mobilization of myelocytes in rabbits.^42^ Yu et al. suggested that VEGF could be defined as a marker of atherosclerosis, based on their experiment in rabbits.^43^ Kimura et al. reported that serum VEGF could be used as a prognostic marker for atherosclerosis development in humans.^44^ In the present study, no relationships were detected between VEGF and atherosclerosis determined by CA-IMT and RRI in RTRs. VEGF inhibits media thickening by accelerating vascular endothelial cell regeneration and improving endothelial function. Milasan et al. reported that VEGF inhibits the inflammatory response, and prevents the progression of atherosclerosis by stimulating the expansion and proliferation of lymphatic vessels and reducing oxidative stress.^45^ Heinonen et al. reported that VEGF causes decreased atherosclerosis development by reducing plasma lipoprotein lipase activity and accumulating chylomicrons, LDL, and triglycerides in large lipoprotein granules.^46^ Lim et al. reported that VEGF and Ang-2 levels increased in diabetic patients, but detected no relationships between them and endothelial damage and atherosclerosis.^47^ Sánchez-Escuredo et al. reported that although an independent relationship was detected between interleukin-8 and C-reactive protein, which are inflammation markers, and increased CA-IMT and CV mortality after renal transplantation, such a relationship was not detected with VEGF.^48^ After renal transplantation, secondary to the decreased atherosclerosis^49^, a decrease in the receptors and effects of angiogenic growth factors synthesized from atherosclerotic plaques may occur. Fiedler et al.^50^ reported that Ang may be responsible for the development of CV events by triggering microinflammatory events on the endothelium without causing atherosclerosis.

The present study has some limitations. First, the study had a cross-sectional design, was conducted in a single center, and included a limited number of patients. Because this study had a small sample size, it was difficult to uncover the traditional and non-traditional risk factors that cause atherosclerosis development. Second, serum Ang-2 and VEGF-A levels and ultrasonographic findings were determined only at the beginning of the study, and follow-up values were not obtained because the study was cross-sectional. Third, no comparisons were made based on serum angiogenic growth factor levels and CA-IMT values in patients who received peritoneal dialysis or hemodialysis treatment before transplantation. Serum angiopoietin and VEGF levels, and atherosclerosis findings were determined only at the beginning of the study, and follow-up values were not obtained. Therefore, the relationship between growth factors and atherosclerosis was not examined in RTRs during the follow-up periods. Additionally, antihypertensive and antihyperlipidemic medications were continued for ethical reasons. Therefore, it is not possible to rule out the direct effects of these drugs on endothelial function. This may have affected the actual atherosclerosis rates in the patients included in this study. Finally, other subtypes of growth factors, tissue receptors, and inflammatory parameters and their effects on atherosclerosis have not been investigated.

## CONCLUSION

Increased serum angiogenic growth factor levels and atherosclerosis development were detected in RTRs. No relationship was detected between the angiogenic growth factors and atherosclerosis. This may be due to decreased levels of serum angiogenic growth factors and receptors synthesized from atherosclerotic plaques that resolved after transplantation. Further multicenter studies with a larger number of patients are needed because of conflicting results.

## References

[1] Sarnak MJ, Levey AS, Schoolwerth AC (2003). Kidney disease as a risk factor for development of cardiovascular disease: a statement from the American Heart Association Councils on Kidney in Cardiovascular Disease, High Blood Pressure Research, Clinical Cardiology, and Epidemiology and Prevention. Circulation.

[2] Recio-Mayoral A, Banerjee D, Streather C, Kaski JC (2011). Endothelial dysfunction, inflammation and atherosclerosis in chronic kidney disease--a cross-sectional study of predialysis, dialysis and kidney-transplantation patients. Atherosclerosis.

[3] Radermacher J, Mengel M, Ellis S (2003). The renal arterial resistance index and renal allograft survival. N Engl J Med.

[4] Chudek J, Kolonko A, Król R (2006). The intrarenal vascular resistance parameters measured by duplex Doppler ultrasound shortly after kidney transplantation in patients with immediate, slow, and delayed graft function. Transplant Proc.

[5] Kramann R, Frank D, Brandenburg VM (2012). Prognostic impact of renal arterial resistance index upon renal allograft survival: the time point matters. Nephrol Dial Transplant.

[6] Shimizu Y, Itoh T, Hougaku H (2001). Clinical usefulness of duplex ultrasonography for the assessment of renal arteriosclerosis in essential hypertensive patients. Hypertens Res.

[7] Calabia J, Torguet P, Garcia I (2014). The relationship between renal resistive index, arterial stiffness, and atherosclerotic burden: the link between macrocirculation and microcirculation. J Clin Hypertens.

[8] Barr MP, Bouchier-Hayes DJ, Harmey JJ (2008). Vascular endothelial growth factor is an autocrine survival factor for breast tumour cells under hypoxia. Int J Oncol.

[9] Shibuya M (2013). Vascular endothelial growth factor and its receptor system: physiological functions in angiogenesis and pathological roles in various diseases. J Biochem.

[10] Yilmaz MI, Sonmez A, Saglam M (2015). A longitudinal study of inflammation, CKD-mineral bone disorder, and carotid atherosclerosis after renal transplantation. Clin J Am Soc Nephrol.

[11] Nafar M, Khatami F, Kardavani B (2007). Atherosclerosis after kidney transplantation: changes of intima-media thickness of carotids during early posttransplant period. Urol J.

[12] Pinho A, Sampaio S, Pestana M (2014). Accelerated atherosclerosis after renal transplantation: an unsuspected cause of uncontrolled hypertension. Int J Nephrol Renovasc Dis.

[13] Junarta J, Hojs N, Ramphul R (2020). Progression of endothelial dysfunction, atherosclerosis, and arterial stiffness in stable kidney transplant patients: a pilot study. BMC Cardiovasc Disord.

[14] Basiratnia M, Fazel M, Lotfi M (2010). Subclinical atherosclerosis and related risk factors in renal transplant recipients. Pediatr Nephrol.

[15] Kasiske BL (1988). Risk factors for accelerated atherosclerosis in renal transplant recipients. Am J Med.

[16] Lindholm A, Albrechtsen D, Frödin L (1995). Ischemic heart disease--major cause of death and graft loss after renal transplantation in Scandinavia. Transplantation.

[17] Turkmen K, Tonbul HZ, Toker A (2012). The relationship between oxidative stress, inflammation, and atherosclerosis in renal transplant and end-stage renal disease patients. Ren Fail.

[18] Heine GH, Gerhart MK, Ulrich C, Köhler H, Girndt M (2005). Renal Doppler resistance indices are associated with systemic atherosclerosis in kidney transplant recipients. Kidney Int.

[19] Akgul A, Sasak G, Basaran C (2009). Relationship of renal resistive index and cardiovascular disease in renal transplant recipients. Transplant Proc.

[20] Brennan DC, Lentine KL (2006). Is there a correlation between atherosclerosis and renal resistive indices in kidney transplant recipients?. Nat Clin Pract Nephrol.

[21] Köger P, Engelberger S, Thalhammer C (2021). Association of intrarenal resistance index and systemic atherosclerosis after kidney transplantation. In Vivo.

[22] David S, Kümpers P, Lukasz A (2010). Circulating angiopoietin-2 levels increase with progress of chronic kidney disease. Nephrol Dial Transplant.

[23] Schmidt RJ, Baylis C (2000). Total nitric oxide production is low in patients with chronic renal disease. Kidney Int.

[24] Yang X, Zhang H, Shi Y (2018). Association of serum angiopoietin-2 with malnutrition, inflammation, atherosclerosis and valvular calcification syndrome and outcome in peritoneal dialysis patients: a prospective cohort study. J Transl Med.

[25] Tsai YC, Lee CS, Chiu YW (2015). Angiopoietin-2 as a prognostic biomarker of major adverse cardiovascular events and all-cause mortality in chronic kidney disease. PLoS One.

[26] David S, John SG, Jefferies HJ (2012). Angiopoietin-2 levels predict mortality in CKD patients. Nephrol Dial Transplant.

[27] Iribarren C, Phelps BH, Darbinian JA (2011). Circulating angiopoietins-1 and -2, angiopoietin receptor Tie-2 and vascular endothelial growth factor-A as biomarkers of acute myocardial infarction: a prospective nested case-control study. BMC Cardiovasc Disord.

[28] Le Dall J, Ho-Tin-Noé B, Louedec L (2010). Immaturity of microvessels in haemorrhagic plaques is associated with proteolytic degradation of angiogenic factors. Cardiovasc Res.

[29] El-Asrar MA, Elbarbary NS, Ismail EA, Bakr AA (2016). Circulating angiopoietin-2 levels in children and adolescents with type 1 diabetes mellitus: relation to carotid and aortic intima-media thickness. Angiogenesis.

[30] Shroff RC, Price KL, Kolatsi-Joannou M (2013). Circulating angiopoietin-2 is a marker for early cardiovascular disease in children on chronic dialysis. PLoS One.

[31] Mayer G (2011). Capillary rarefaction, hypoxia, VEGF and angiogenesis in chronic renal disease. Nephrol Dial Transplant.

[32] Ahmed A, Fujisawa T (2011). Multiple roles of angiopoietins in atherogenesis. Curr Opin Lipidol.

[33] David S, Kümpers P, Hellpap J (2009). Angiopoietin 2 and cardiovascular disease in dialysis and kidney transplantation. Am J Kidney Dis.

[34] Blann AD, Belgore FM, McCollum CN (2002). Vascular endothelial growth factor and its receptor, Flt-1, in the plasma of patients with coronary or peripheral atherosclerosis, or Type II diabetes. Clin Sci.

[35] Liu Y, Hong K, Weng W (2023). Association of vascular endothelial growth factor (VEGF) protein levels and gene polymorphism with the risk of chronic kidney disease. Libyan J Med.

[36] Nguyen TTU, Kim H, Chae YJ, Jung JH, Kim W (2022). Serum VEGF-D level is correlated with renal dysfunction and proteinuria in patients with diabetic chronic kidney disease. Medicine.

[37] Pilmore HL, Eris JM, Painter DM (1999). Vascular endothelial growth factor expression in human chronic renal allograft rejection. Transplantation.

[38] Rintala SE, Savikko J, Rintala JM, von Willebrand E (2006). Vascular endothelial growth factor (VEGF) ligand and receptor induction in rat renal allograft rejection. Transplant Proc.

[39] Felmeden DC, Spencer CG, Belgore FM (2003). Endothelial damage and angiogenesis in hypertensive patients: relationship to cardiovascular risk factors and risk factor management. Am J Hypertens.

[40] Celletti FL, Waugh JM, Amabile PG (2001). Vascular endothelial growth factor enhances atherosclerotic plaque progression. Nat Med.

[41] Inoue M, Itoh H, Ueda M (1998). Vascular endothelial growth factor (VEGF) expression in human coronary atherosclerotic lesions: possible pathophysiological significance of VEGF in progression of atherosclerosis. Circulation.

[42] Ohtani K, Egashira K, Hiasa K (2004). Blockade of vascular endothelial growth factor suppresses experimental restenosis after intraluminal injury by inhibiting recruitment of monocyte lineage cells. Circulation.

[43] Yu ZM, Deng XT, Qi RM (2018). Mechanism of chronic stress-induced reduced atherosclerotic medial area and increased plaque instability in rabbit models of chronic stress. Chin Med J.

[44] Kimura K, Hashiguchi T, Deguchi T (2007). Serum VEGF--as a prognostic factor of atherosclerosis. Atherosclerosis.

[45] Milasan A, Smaani A, Martel C (2019). Early rescue of lymphatic function limits atherosclerosis progression in Ldlr-/- mice. Atherosclerosis.

[46] Heinonen SE, Kivelä AM, Huusko J (2013). The effects of VEGF-A on atherosclerosis, lipoprotein profile, and lipoprotein lipase in hyperlipidaemic mouse models. Cardiovasc Res.

[47] Lim HS, Lip GY, Blann AD (2005). Angiopoietin-1 and angiopoietin-2 in diabetes mellitus: relationship to VEGF, glycaemic control, endothelial damage/dysfunction and atherosclerosis. Atherosclerosis.

[48] Sánchez-Escuredo A, Pastor MC, Bayés B (2010). Inflammation, metalloproteinases, and growth factors in the development of carotid atherosclerosis in renal transplant patients. Transplant Proc.

[49] Yilmaz MI, Sonmez A, Saglam M (2015). A longitudinal study of inflammation, CKD-mineral bone disorder, and carotid atherosclerosis after renal transplantation. Clin J Am Soc Nephrol.

[50] Fiedler U, Reiss Y, Scharpfenecker M (2006). Angiopoietin-2 sensitizes endothelial cells to TNF-alpha and has a crucial role in the induction of inflammation. Nat Med.

